# Percutaneous Tibiotalocalcaneal Nailing in Fragility Ankle Fractures of the Elderly: A Single-Center Case Series

**DOI:** 10.7759/cureus.99592

**Published:** 2025-12-19

**Authors:** Vishal Upadhyay, André Fernandes, Smit Shah, Heath Taylor, Mark Farrar

**Affiliations:** 1 Trauma and Orthopaedics, York and Scarborough Teaching Hospitals NHS Foundation Trust, York, GBR; 2 Trauma and Orthopaedics, Poole Hospital, Poole, GBR

**Keywords:** ankle and foot, ankle fractures, early weight-bearing, elderly, foot and ankle surgery, fragility, fragility ankle fracture, fragility fractures, osteoporotic bone, tibiotalocalcaneal nailing

## Abstract

Background: Fragility ankle fractures in elderly patients are associated with high rates of wound complications, fixation failure, and prolonged loss of mobility. For individuals with significant osteopenia or compromised soft tissues, conventional open reduction and internal fixation may be unsuitable. Retrograde tibiotalocalcaneal (TTC) nailing provides a minimally invasive, load-sharing construct that facilitates early weight-bearing. This study reports outcomes from a retrospective single-center case series evaluating percutaneous TTC nailing in elderly patients with unstable fragility ankle fractures.

Methods: A retrospective review was performed on all consecutive patients aged ≥65 years who underwent percutaneous TTC nailing at a UK trauma unit between February 2007 and February 2008. Demographic data, fracture classification, complications, radiographic union, Olerud-Molander Ankle Score (OMAS), mobility status, and mortality were recorded. Radiographic union was defined as bridging callus across ≥3 cortices.

Results: Twenty-three patients (mean age 89 years; 22 female) were included. Arbeitsgemeinschaft für Osteosynthesefragen* *(AO) fracture types included 44-B2 (n=10), 44-B3 (n=9), 44-C2 (n=2), 43-A1 (n=1), and 43-A3 (n=1). The median hospital stay was nine days. Two superficial infections occurred; no deep infections, thromboembolic events, or implant failures were recorded. Most surviving patients demonstrated radiographic union by 12 weeks. The mean OMAS was 49.7±8.5. Thirty-day and one-year mortality rates were 21.7% and 43.5%, respectively.

Conclusions: This study reports descriptive outcomes of a small retrospective case series and does not allow causal inference or comparison with alternative treatments. Within these limitations, percutaneous TTC nailing provided stable fixation with low observed wound-related complications in this frail, elderly cohort. The findings should be interpreted cautiously, and prospective comparative studies are required.

## Introduction

Ankle fractures are common fragility injuries in older adults, frequently associated with reduced independence, prolonged hospitalisation, and high complication rates [1]. Traditional open reduction and internal fixation may be unsuitable for elderly individuals with severe osteopenia, multiple comorbidities, or compromised soft-tissue envelopes, as these factors substantially increase the risks of wound breakdown and fixation failure [2,3].

Retrograde tibiotalocalcaneal (TTC) nailing provides a minimally invasive fixation option that spans the ankle and subtalar joints, creating a load-sharing construct capable of tolerating early weight-bearing in patients with limited functional demand [4-7]. The technique is especially advantageous where soft-tissue preservation is a priority.

The primary objective of this study was to describe the clinical and radiographic outcomes of consecutive elderly patients treated with percutaneous TTC nailing for unstable fragility ankle fractures. Secondary objectives included documenting complications, radiographic union at 12 weeks, mortality at defined intervals, and functional status using the Olerud-Molander Ankle Score (OMAS) at the final follow-up. A minimum clinical follow-up of 12 weeks was required for inclusion.

## Materials and methods

Study design and setting

This retrospective single-center case series included all consecutive patients aged 65 years or older who underwent percutaneous TTC nailing for unstable ankle or distal tibial fractures at York Hospital, York, England, UK. The study period reflects the introduction of TTC nailing for fragility ankle fractures at our institution and captures the first consecutive cohort treated with this technique.

Patients were identified through theatre logbooks, operative records, and electronic medical documentation. All individuals listed for hindfoot TTC nailing between February 2007 and February 2008 were screened (n=30). Eligibility required age of at least 65 years, an unstable ankle or distal tibial fracture occurring within 4 cm of the ankle joint, radiographic osteopenia or clinically significant soft-tissue compromise documented by the treating surgeon, and a minimum clinical follow-up of 12 weeks. After record review, four patients were excluded because of incomplete documentation and three for being revision procedures for failed primary open reduction and internal fixation. A final cohort of 23 patients met all inclusion criteria, representing the entirety of consecutive eligible cases treated during the study period. Fractures were included only when conventional open reduction and internal fixation was considered unsuitable due to osteopenia or soft-tissue insufficiency, and patients were excluded if pathological fractures, previous hindfoot fusion, or insufficient clinical or radiographic data were present.

The primary outcome was radiographic union at 12 weeks. Radiographic union was defined as bridging callus across at least three cortices, acknowledging the potential limitations of this criterion in the context of TTC constructs designed to immobilise the hindfoot. Secondary outcomes included surgical and medical complications according to predefined criteria; mortality at in-hospital, 30-day, three-month, six-month, and 12-month intervals; functional outcome assessed using the OMAS at the final follow-up; and return to pre-injury mobility status. Return to pre-injury mobility was defined as the patient achieving the same mobility category, independent household ambulation, assisted household ambulation, or non-ambulatory, documented in the admission clerking notes. Venous thromboembolism events were identified through electronic medical records, discharge summaries, radiology reports, and follow-up clinic documentation. The OMAS was administered at the final available clinic review, typically at 12 weeks.

This project was registered with the institutional clinical governance office as a retrospective service evaluation.

Surgical technique

All procedures were performed by consultant orthopaedic trauma surgeons experienced in TTC nailing or by middle-grade surgeons under direct consultant supervision. Anaesthesia was either general or regional, determined by anaesthetist and patient preference. Antibiotic prophylaxis was administered in accordance with departmental protocols at the time. Early in the study period, this consisted of cefuroxime 1.5 g at induction, followed by two postoperative doses of 750 mg at eight and 16 hours. Later, the regimen was amended to a single perioperative dose of flucloxacillin 1 g combined with gentamicin 80 mg. All patients were positioned supine on a radiolucent table in a laminar-flow theatre. No tourniquet was used.

After sterile preparation and draping, closed reduction was achieved under fluoroscopic guidance to ensure satisfactory alignment in the anteroposterior and lateral planes. A 2 cm midline plantar incision was made at the junction of the middle and anterior thirds of the heel pad. Blunt dissection proceeded to the plantar surface of the calcaneus, with the careful medial retraction of the lateral plantar neurovascular bundle. The entry guide was introduced through this incision and seated on the plantar calcaneal cortex with gentle mallet taps.

With the ankle maintained in a plantigrade position, a threaded guide pin was advanced through the entry guide using a power driver, crossing the calcaneus and talus before entering the tibial medullary canal. Fluoroscopy was used continuously to confirm the maintenance of fracture reduction, neutral alignment of the ankle and subtalar joints, and central placement of the guide pin within the tibia. A cannulated awl was then used to prepare the entry point, followed by initial reaming over the guide pin to the level of the tibial metaphysis. The threaded guide pin was subsequently exchanged for a ball-tipped guidewire, and flexible cannulated reamers were used to ream the tibia to 0.5-1 mm above the intended nail diameter.

Available nail diameters were 10 mm and 12 mm, each in lengths of 150 mm, 200 mm, and 250 mm. The correct nail length was determined using a radiographic trial sizer. The selected nail was mounted onto the targeting jig and introduced over the guidewire with gentle malleting, positioning the three distal locking screw holes to allow optimal fixation in both the talus and calcaneus. This configuration typically permitted the insertion of two screws into the calcaneus and one into the talus. Distal locking was usually performed from lateral to medial, although a posterolateral-to-anteromedial trajectory was occasionally selected to maximise screw purchase and alignment with the talar axis. Distal screws measured 5.5 mm and were self-tapping.

The distal end of the nail was positioned flush with, or slightly recessed within, the calcaneal cortex. In patients with pes planus, a nail tip projecting less than 5 mm above the cortex was considered acceptable provided it did not extend below the plantar weight-bearing surface. After confirming distal fixation under fluoroscopy, proximal locking was performed from medial to lateral using two static 4.5 mm self-tapping screws inserted via the jig. Following the removal of the jig, an end cap was applied. End caps were either non-impinging or impinging types designed to engage a distal screw to reduce the risk of screw migration. Although the nail system includes mechanisms for joint compression and dynamisation used in hindfoot arthrodesis, these were not employed in this cohort, as treatment aimed to stabilise fractures rather than achieve formal fusion.

Postoperative protocol

All patients were permitted to weight-bear as tolerated immediately following surgery, using either a controlled ankle motion boot or a walking cast for six weeks. Follow-up was routinely undertaken at two, six, and 12 weeks, with additional review arranged according to clinical progress. Standardised orthogonal radiographs were obtained at each clinic visit to evaluate alignment, consolidation, and implant position.

Data collection and outcome measures

Demographic characteristics, comorbidities, Arbeitsgemeinschaft für Osteosynthesefragen (AO) fracture classification, open fracture grade, complications, radiographic outcomes, and mortality at predefined intervals (in-hospital, 30-day, three-month, six-month, and 12-month) were recorded from the electronic patient record. Functional outcomes were assessed using the OMAS, calculated at the final follow-up in accordance with the original description by Olerud and Molander [8]. The OMAS ranges from 0 (complete impairment) to 100 (normal function) and is typically interpreted as excellent (91-100), good (61-90), fair (31-60), or poor (0-30). Radiographic union was defined as bridging callus across at least three cortices on orthogonal radiographs.

Ethical considerations

This study used fully anonymised retrospective data and was deemed exempt from formal ethics review under NHS Health Research Authority guidelines. Consent for publication of de-identified radiographs was obtained when required.

## Results

Twenty-three patients met the inclusion criteria. The mean age was 89 years, reflecting a notably frail and elderly cohort, and 22 of the 23 patients were female. Fracture patterns encompassed a range of AO classifications, including 44-B2, 44-B3, 44-C2, 43-A1, and 43-A3 injuries. Four fractures were open, of which three were classified as Gustilo-Anderson grade IIIB. The median length of hospital stay was nine days (interquartile range (IQR) 6-16), although individual stay durations varied according to medical comorbidity and postoperative recovery (Table 1).

**Table 1:** Patient demographics and fracture characteristics All patients met the criteria for fragility fracture with radiographic osteopenia. AO: Arbeitsgemeinschaft für Osteosynthesefragen; Gustilo IIIB: Gustilo-Anderson grade IIIB open fracture

Variable	Value
Age, years (mean±SD)	89±7
Female sex, n (%)	22 (95.7)
Male sex, n (%)	1 (4.3)
AO classification	
44-B2, n (%)	10 (43.5)
44-B3, n (%)	9 (39.1)
44-C2, n (%)	2 (8.7)
43-A1, n (%)	1 (4.3)
43-A3, n (%)	1 (4.3)
Open fracture grade	
Closed, n (%)	19 (82.6)
Gustilo IIIB, n (%)	3 (13)
Other grade, n (%)	1 (4.3)

Clinical outcomes

Two of 23 patients (8.7%) developed superficial surgical site infections, both of which resolved with oral antibiotic therapy. No deep infections occurred, and there were no cases of implant failure or clinically significant malalignment on follow-up radiographs. No venous thromboembolic events were identified through review of medical records, discharge summaries, radiology reports, or outpatient documentation.

Radiographic union was achieved in 22 of 23 patients (95.7%), defined as bridging callus across at least three cortices at 12 weeks.

Follow-up data at 12 weeks were available for 22 of 23 patients (95.7%). OMAS was recorded in 22 surviving patients at the final follow-up, representing complete functional outcome capture among survivors.

Mortality was substantial and reflected the extreme age and systemic frailty of the cohort rather than procedure-specific complications. In-hospital mortality was 13% (3/23), rising to 21.7% (5/23) at 30 days and 43.5% (10/23) at one year (Table 2). Mortality rates were not adjusted for comorbid burden due to sample size limitations.

**Table 2:** Clinical outcomes and complications Radiographic union is defined as bridging callus across ≥3 cortices at 12 weeks. Mortality rates reflect systemic frailty typical of geriatric trauma cohorts rather than procedure-related complications. IQR: interquartile range

Outcome measure	Data
Superficial infection	2 (8.7%)
Deep infection	0 (0%)
Thromboembolic events	0 (0%)
Implant failure	0 (0%)
Nail-site pain	2 (8.7%)
Radiographic union	22/23 (95.7%)
Mean Olerud-Molander Ankle Score (points)	49.7±8.5
Return to pre-injury mobility	18 (78.3%)
In-hospital mortality	3 (13%)
30-day mortality	5 (21.7%)
1-year mortality	10 (43.5%)
Length of stay, days (median (IQR))	9 (6-16)

Functional outcomes

**Figure 1:**
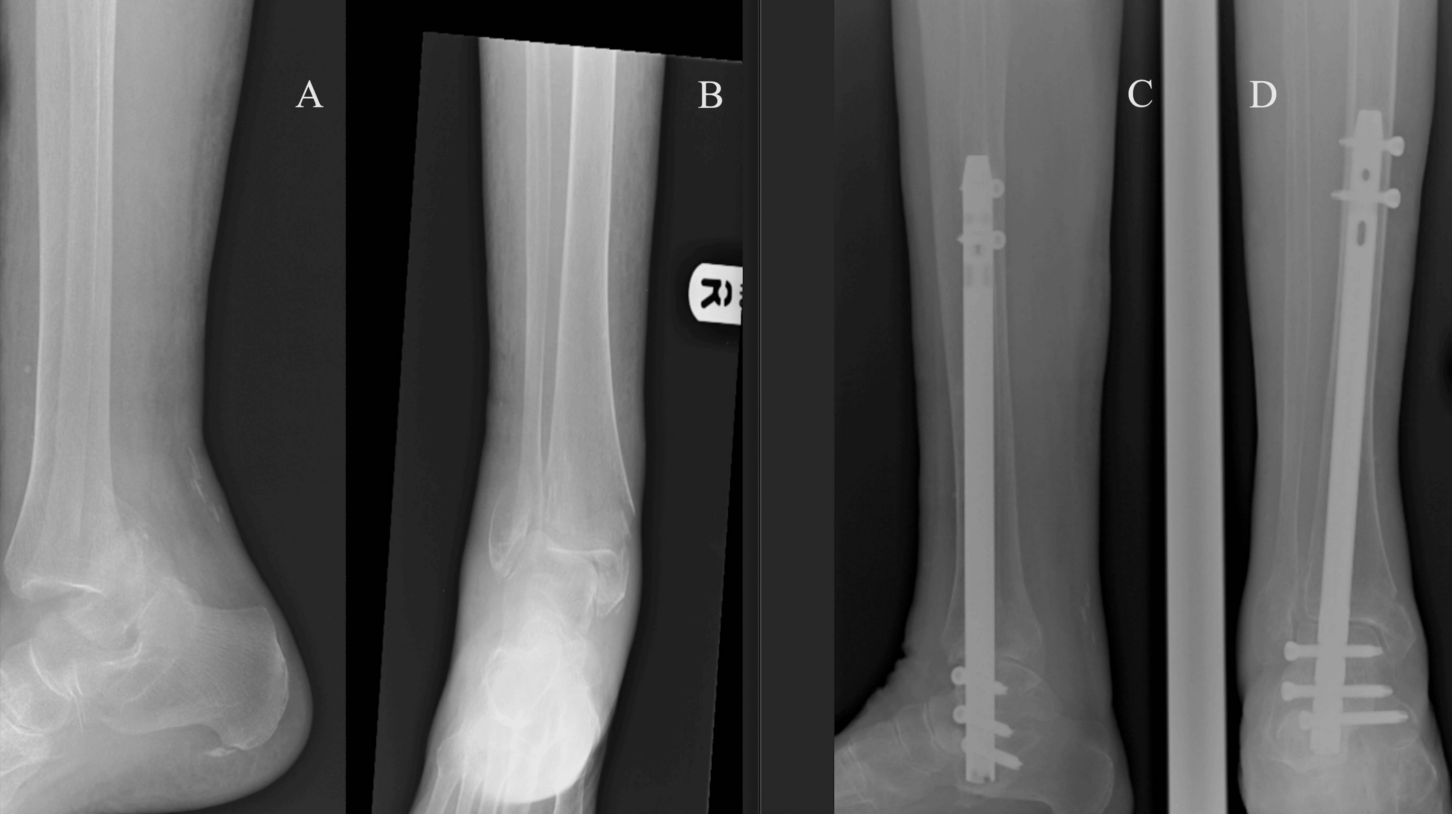
Sequential anteroposterior and sagittal radiographs demonstrating the management of a fragility ankle fracture using percutaneous retrograde TTC nailing in an elderly patient with osteoporotic bone (A) Preoperative lateral (sagittal) view showing an unstable osteoporotic ankle fracture with soft-tissue compromise. (B) Preoperative anteroposterior view confirming fracture displacement and osteopenic bone architecture. (C) Postoperative lateral (sagittal) view demonstrating stable reduction and fixation with a TTC nail spanning the tibiotalar and subtalar joints. (D) Postoperative anteroposterior view showing satisfactory alignment and distal interlocking screw fixation. TTC: tibiotalocalcaneal

Functional assessment demonstrated a mean OMAS of 49.7±8.5 at the final available follow-up (Table 3). This score, recorded at a single postoperative time point, should be interpreted within the context of TTC fusion, which eliminates ankle and subtalar motion and inherently limits functional scores compared with joint-preserving treatment. Despite these constraints, 18 of 23 patients (78.3%) returned to their documented pre-injury mobility category, typically household or short-distance ambulation with aids (Figure 1).

**Table 3:** Summary of patient demographics, fracture characteristics, pre-injury and follow-up mobility, complications, mortality, fracture union, pain at follow-up, and functional outcome (OMAS) following the surgical fixation of distal tibial fractures pre-/post-op: pre-/postoperative; FU: follow-up; #: fracture; COPD: chronic obstructive pulmonary disease; N: no; Y: yes; ARF: acute renal failure; LVF: left ventricular failure; CA: carcinoma; PVD: peripheral vascular disease; MI: myocardial infarction; prefix 44: malleolar fractures in AO classification; prefix 43: distal tibial and fibular fractures in AO classification; OMAS: Olerud-Molander Ankle Score; VAC: vacuum-assisted closure; AKA: above-knee amputation; AO: Arbeitsgemeinschaft für Osteosynthesefragen fracture classification; Gr: Gustilo-Anderson grade

S. no.	Age (yrs)	Fracture pattern (AO)	Pre-injury mobility	Follow-up mobility	Reason for decreased mobility	Death (weeks post-op)	Complications	Pain at FU	Union	OMAS
1	88	Gr IIIB, open 44-C2	Frame	NA	NA	9 days: pneumonia	Worsened COPD, pneumonia	N	NA	NA
2	95	44-B2	Stick	Stick	NA	NA	Pain at the nail insertion site (steroid injection)	Y, relieved	Y	55
3	93	44-B2	Frame	Frame	NA	NA	Nil	N	Y	55
4	89	44-B2	Frame	Frame	NA	NA	Nil	N	Y	55
5	84	44-B3	Independent	Independent	NA	NA	Wound infection, screw and nail removal	N	Y	55
6	91	Supramalleolar # 43-A1	Wheelchair/hoisted	Wheelchair	NA	33 weeks: unrelated	Nil	N	Y	50
7	89	44-B2	Frame	Frame	NA	47 weeks	Nil	N	Y	50
8	87	44-B3	Frame	-	NA	6 days: ARF/LVF	ARF, decompensated LVF	N	NA	NA
9	98	44-B2	Frame	Frame	NA	97 weeks: unrelated	Nil	N	Y	55
10	91	Gr IIIA, open 44-C2	Frame	Frame	NA	46 weeks: unrelated	Pain at the nail insertion site	Y, coping	Y	25
11	89	44-B2	Frame	Hoisted	Severe dementia and poor cognition	15 weeks: unrelated	Pre-op skin necrosis (VAC)	N	Y	50
12	67	44-B3	Independent	Independent	NA	4 weeks: CA metastasis	Nil	N	NA	70
13	88	44-B2	Frame	Frame	NA	NA	Superficial infection (antibiotics)	N	Y	45
14	87	Supramalleolar # 43-A3	Frame	Frame	NA	NA	Nil	N	Y	55
15	101	44-B3	Frame	Frame	NA	56 weeks: unrelated	Nil	N	N	55
16	79	44-B3	Wheelchair/frame	NA	NA	9 weeks: pneumonia	Worsened PVD, gangrene → AKA	NA	NA	NA
17	83	44-B2	Independent	Frame	Frailty, confusion	NA	Nil	N	Y	40
18	94	Gr IIIB, open 44-B2	Frame	Frame	NA	NA	Nil	N	Y	55
19	99	44-B3	Frame	Frame	NA	NA	Nil	N	Y	40
20	97	44-B3	Independent	Frame	Frailty, age	NA	Nil	N	Y	50
21	95	44-B2	Frame	Frame	NA	4 weeks: unrelated	Nil	N	NA	50
22	84	Gr IIIA, open 44-B3	Independent	Frame	Open wounds slow to heal	NA	Wounds healed after debridement	N	Y	35
23	84	44-B3	Wheelchair	NA	NA	8 days: post-op MI	Nil	N	NA	NA

Follow-up

Follow-up data at 12 weeks were available for 22 of 23 patients (95.7%). OMAS was recorded in 22 surviving patients at the final follow-up. 

## Discussion

This study reports the outcomes of percutaneous TTC nailing for unstable fragility ankle fractures in a very elderly and medically complex cohort. The patients included in this series exhibited pronounced osteopenia, vulnerable soft tissues, and markedly limited physiological reserve. In this context, percutaneous TTC nailing appeared to offer a method of stabilisation that avoided the wound complications commonly associated with traditional open approaches. The low incidence of superficial wound problems, together with the absence of deep infection and implant failure, is noteworthy given the high medical frailty of the group. These observations should be interpreted cautiously, as the study design does not allow inferences regarding effectiveness or superiority.

The finding that many surviving patients achieved their pre-injury level of mobility suggests that the procedure may provide a practical means of facilitating early protected weight-bearing in individuals who are unsuitable for conventional open reduction and internal fixation. Early mobilisation is an established principle of geriatric trauma care, and TTC constructs are designed to provide immediate hindfoot stability in low-demand patients, which may help support this goal [9]. Despite this, the absence of a comparative cohort prevents any definitive assessment of whether TTC nailing offers advantages over alternative management strategies, including both open fixation and conservative treatment.

Mortality in this series was high, reflecting the substantial frailty and comorbidity burden of the population. The observed rates align with those reported for similarly vulnerable geriatric trauma groups, including individuals with hip fractures [10-15]. It is therefore likely that mortality predominantly reflects underlying systemic frailty rather than complications arising from the fixation technique. The study period corresponds to the early adoption of TTC nailing for fragility ankle fractures at our institution, which explains the limited sample size and the historical nature of the dataset.

When considered collectively, the findings of this case series contribute descriptive information regarding the use of TTC nailing in extremely frail elderly patients with unstable fragility ankle fractures. The technique provides stable fixation, protects soft tissues, and permits early loading, which are features consistent with the principles of orthogeriatric practice [16]. However, the limitations of the study require that these findings be regarded as observational rather than evaluative.

Strengths and limitations

A strength of the study is its inclusion of a consecutive series of very elderly patients treated with a consistent percutaneous technique, thereby offering insight into real-world practice in a population characterised by profound osteopenia, multimorbidity, and minimal functional reserve. This represents a group in whom traditional open fixation is often contraindicated.

This study has significant limitations. The retrospective design, small sample size, historical study period, and absence of a comparator group introduce substantial risk of selection bias and prevent any causal inference. The inclusion criteria relied on clinical judgement, which may affect reproducibility. The OMAS was employed as a pragmatic measure of function, although it is not validated for fused hindfoot constructs and must therefore be interpreted cautiously. The OMAS is a self-reported outcome measure developed specifically for patients following ankle fracture and includes items such as running, jumping, and squatting, which presuppose a mobile ankle joint. Its use in cohorts treated with TTC constructs that intentionally immobilise the ankle-subtalar complex therefore has conceptual limitations [17-20].

Radiographic union was defined pragmatically as bridging callus across ≥3 cortices on plain radiographs. However, standard radiographs have been shown to overestimate fusion compared with CT in hindfoot arthrodesis, and there is no universally accepted gold-standard definition of union in foot and ankle fusion procedures; CT-based assessments demonstrate lower and more variable union rates than suggested by plain films [21-23].

The retrospective, single-center design and the need for patients to survive and engage with follow-up introduce a high risk of selection bias, a recognised problem in orthogeriatric and ageing research. Similar hip fracture cohorts often exclude the most cognitively impaired or acutely unwell patients or preferentially include either the "healthiest" or the "sickest" subsets, which substantially limits generalisability and may distort mortality and complication estimates [24-27].

## Conclusions

This retrospective descriptive case series provides preliminary observational data on percutaneous TTC nailing for fragility ankle fractures in a very elderly, low-demand population. Within the limitations of the study design, TTC nailing appeared to offer a stable construct with low observed wound-related complications. These findings should be interpreted cautiously due to significant methodological limitations. Prospective, adequately powered comparative studies are required to establish the role of TTC nailing relative to conventional fixation or conservative management.
